# Gender-specific difference in the recurrence of flexion contracture after total knee arthroplasty

**DOI:** 10.1186/s40634-021-00409-z

**Published:** 2021-10-06

**Authors:** Tomofumi Kinoshita, Kazunori Hino, Tatsuhiko Kutsuna, Kunihiko Watamori, Takashi Tsuda, Hiromasa Miura

**Affiliations:** Department of Orthopedic Surgery, Ehime University Graduate School of Medicine, Shitsukawa, Toon, Ehime 791-0295 Japan

**Keywords:** Total knee arthroplasty, Flexion contracture, Hyperextension, Navigation system

## Abstract

**Background:**

Range of motion after total knee arthroplasty (TKA) can impact patients’ daily lives. Nevertheless, flexion contracture (FC) often recurs after TKA, even upon achieving full extension intraoperatively. This study aimed to evaluate the relationship among preoperative, intraoperative, and postoperative knee extension angles, and clarify the risk factor for postoperative FC.

**Methods:**

One hundred forty-seven knees undergoing TKA using a navigation system were evaluated. We measured the pre- and postoperative (6 months after TKA) extension angles using a goniometer, and intraoperative (before and after TKA) extension angle using a navigation system; the correlation between these angles at each time point was evaluated.

**Results:**

The mean preoperative, intraoperative (before and after TKA) and postoperative extension angles were -9.9°, -6.8°, -0.1°, and -2.0°. Regarding intraoperative extension angle after TKA, 58 knees showed ≤ 5° hyperextension and six knees showed > 5° hyperextension. At 6 months, no cases showed hyperextension and 105 knees showed full extension. The mean intraoperative extension angle after TKA in the postoperative full extension group was 0.4°. A significant correlation was found among extension angles at each point (*p*<0.01, respectively). However, the intraoperative extension angle after TKA correlated with the postoperative extension angle only in females. Contrarily, the recurrence rate of FC was significantly higher in males than in females (*p*<0.01).

**Conclusion:**

Intraoperative extension angles significantly correlated with pre- and postoperative extension angles in TKA. Moreover, intraoperative mild (≤ 5°) hyperextension is acceptable for postoperative full extension. There was a gender-specific difference in correlation between intra- and postoperative knee extension angles.

**Level of evidence:**

III.

## Background

Total knee arthroplasty (TKA) is an effective surgical treatment for severe osteoarthritis. Previous studies have proved the effectiveness of TKA in improving alignment, pain, and functional status. Nevertheless, 15–30% of patients are unsatisfied with their outcomes [2, 7]. Among various factors that influence the clinical results, the range of motion (ROM) after TKA is a critical factor for patient satisfaction [21, 30, 31]. Flexion contracture is the common problem for postoperative knee, causing clinical symptoms [3, 5]. In TKA, surgeons aim to accomplish intraoperative full knee extension, whereas recurrence in flexion contracture is often experienced after TKA surgery even when surgeons confirm intraoperative full knee extension. Previous studies have already demonstrated the negative effect of flexion contracture on daily movement [30, 31]. Thus, many researchers studied the surgical technique for avoiding postoperative flexion contracture. The intraoperative extension gap is related to postoperative knee extension angle, demonstrating the importance of post-implantation extension laxity [24]. Additional femoral bone cutting was proven to improve intraoperative flexion contracture [17]. Moreover, other papers described the surgical method of soft-tissue release and reported the efficacy of posterior capsular release for flexion contracture [22]. However, little study has been done about the optimal intraoperative knee extension angle under anaesthesia. In TKA, surgeons take a subjective assessment by themselves regarding whether full extension has accomplished intraoperatively. There is no clear indicator of intraoperative knee extension angle. Therefore, to avoid recurrence in flexion contracture, a precise objective index of optimal intraoperative knee extension angle is needed.

Moreover, we should be deliberate about the temporal changes in knee extension angle after TKA, and clarify the factors influencing postoperative flexion contracture. Our study focused on the influence of intraoperative knee extension angle on postoperative knee extension angle. Recent advancements in intraoperative analysis technology including a navigation system have enabled more accurate intraoperative evaluation of the knee [9, 10, 14, 16]. We assessed the intraoperative knee extension angle in TKA using a navigation system for accurate evaluation. We measured the knee extension angle at four time points: preoperative knee extension angle without anaesthesia, intraoperative knee extension angle before TKA under anaesthesia, intraoperative knee extension angle after TKA under anaesthesia (after skin closure), and postoperative knee extension angle 6 months after TKA.

We aimed to 1) evaluate the relationship among preoperative, intraoperative, and postoperative knee extension angles; 2) assess the influence of intraoperative hyperextension on postoperative knee extension angle; and 3) investigate the impact of pre- and intraoperative flexion contracture for postoperative residual flexion contracture.

## Materials and methods

We analysed 151 knees in 141 Japanese patients with osteoarthritis with either posterior-stabilised (PS) or bi-cruciate-stabilised (BCS) TKA (Persona PS, LPS-flex: Zimmer, Warsaw, IN, USA, Journey II BCS: Smith & Nephew, London, UK). All procedures were performed using measured resection techniques with a navigation system (Precision Knee Navigation Software version 4.0, Stryker, Kalamazoo, MI, USA). To accurately assess and minimise the influence of clinical variables, patients with a history of knee surgery (*n* = 1) and valgus deformities (*n* = 3) were excluded. All patients had been diagnosed with medial knee osteoarthritis with varus deformities. The patient population comprised 112 females and 35 males (mean age: 74.9 ± 6.6 years, range: 49–89 years), with normal distribution. Table 1 shows the preoperative patients’ characteristics.

**Table 1:** Preoperative patient characteristics

	Total Mean ± SD (range)	Male Mean ± SD (range)	Female Mean ± SD (range)	*p* value
Number of patients	147	35	112	
Age (years)	74.9 ± 6.6	76.0 ± 7.2	74.6 ± 6.5	0.16
HKA angle (°)	12.3 ± 5.6 (3–32)	13.1 ± 4.0 (5–19)	12.0 ± 5.9 (3–32)	0.20
Maximum extension (°)	-9.9 ± 6.9 (-35-0)	-9.2 ± 6.8 (-25-0)	-10.1 ± 7.1 (-35-0)	0.48
Maximum flexion (°)	117.1 ± 15.4 (80-145)	124.9 ± 11.5 (90–140)	114.6 ± 15.7 (80–145)	0.01*

*SD* standard deviation, *HKA angle* Hip-knee-ankle angle

*Significantly different among genders (*p*<0.05)

### Methods

**Fig. 1:**
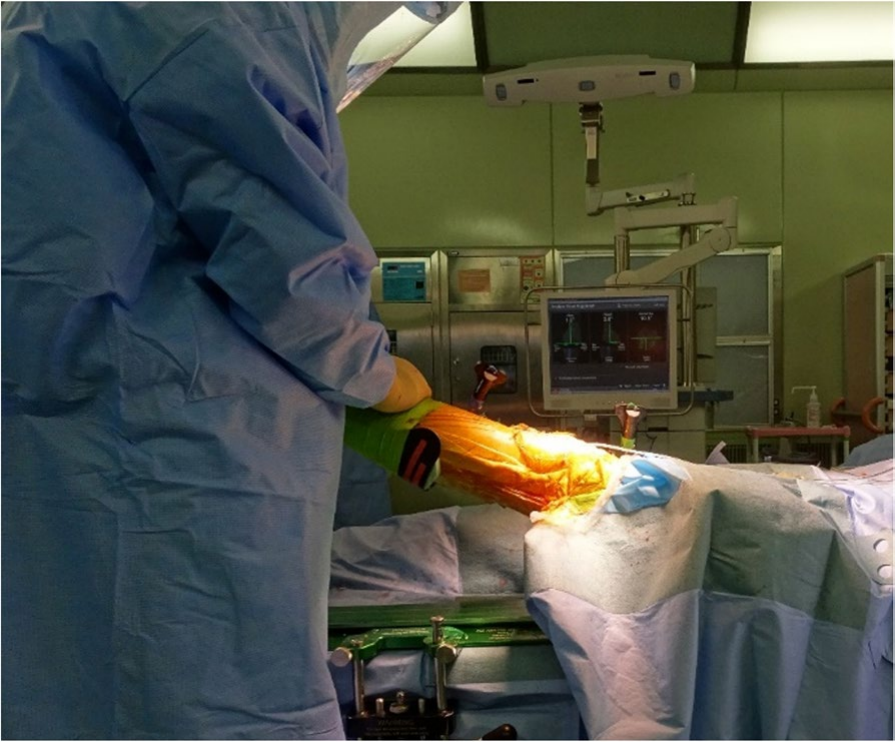
The measurement of intraoperative maximum knee extension angle under passive knee extensional stress

The air tourniquet was inflated (250 mm Hg) with patients under general anaesthesia. The registration was performed according to the instructions of the navigation system. First, a skin incision was made to expose the subcutaneous tissue. We investigated the centre of the hip using infrared signal transducers during hip rotation avoid pelvic movement. Thereafter, the centre of the knee and ankle were registered based on the following: 1) the centre of the distal femur, 2) the surface of the distal femur, 3) the anterior side of the distal femur, 4) the centre of the proximal tibia, 5) the surface of the proximal tibia, 6) the medial malleolus, and 7) the lateral malleolus. Registration was performed with osteophytes and soft tissues, with anterior cruciate ligament preservation. The femoral and tibial anteroposterior and rotational axes were identified using their anatomical landmarks. After registration, the joint capsule was temporarily closed using four suture strands. Maximum passive knee extension stress was manually applied without angular acceleration. Passive extension stress was applied while supporting the foot with an open palm and placing the other hand on the anterior side of the tibia. Under passive stress, the maximum knee extension angle was measured using the navigation system (Fig. 1).

Following this, the distal femur was cut perpendicular to its mechanical axis of the femur using the navigation system with a measured resection technique, and the proximal tibial cut was made perpendicular to the mechanical axis of the tibia based on the concept of mechanical alignment. Regarding tibial alignment in the sagittal plane, we aimed for a 3° posterior tibial slope. Besides, the tibial component rotational angle was aimed to be parallel to the line connecting one-third of the tibial tubercle to the centre of the cut surface. To determine the rotational angle of the femoral component, we used the surgical transepicondylar axis as the index of femoral rotation. Before the operation, we calculated the gap of angle between the surgical transepicondylar axis and the posterior condylar axis on the axial view of computed tomography scans to decide the rotational angle. After osteophyte removal, we placed trial components and a trial insert. We typically began with the thinnest insert in these TKA procedures. Then, we evaluated knee stability using a manual varus–valgus test throughout extension to deep flexion in that condition. We increased the insert size in cases showing excessive medial laxity or excessive flexion laxity. Conversely, in cases with flexion contracture or with inappropriate soft-tissue balance, the posterior knee capsule, the medial collateral ligament, or other tissues were carefully and selectively released for optimal soft-tissue balance throughout the ROM [13]. After the trial, proper thickness components and inserts were placed in the appropriate position with cement, and the surgical incision was closed. Subsequently, the same procedure performed for evaluating maximum knee extension angle before TKA was repeated. The maximum knee extension angle under passive extensional stress after TKA was recorded. The test-retest reliability of the intraoperative maximum extension angle under passive extensional stress indicated that interclass and intraclass correlation coefficients (ICCs) were sufficiently high, with values of > 0.9. Table 2 shows the postoperative implant alignment after TKA. There was no deviation case in the present study. Regarding the postoperative rehabilitation, all patients receive physical therapy and rehabilitation until at least 6 weeks after TKA. Mild passive knee extension exercises were performed during early rehabilitation.

**Table 2:** Postoperative HKA-angle and implant sagittal alignment

	Total (***n*** = 147)	Male (***n*** = 35)	Female (***n*** = 112)	*p* value
	Mean ± SD	Mean ± SD	Mean ± SD	
HKA-angle	0.7±2.3°	0.9±0.4°	0.7±0.2°	0.73
Femoral component sagittal alignment	4.2±2.7° (flexion)	4.9±0.5° (flexion)	4.0±0.3° (flexion)	0.09
Posterior tibial slope	3.7±2.2°	3.0±0.4°	3.8±0.2°	0.08

*SD* standard deviation, *HKA angle* Hip-knee-ankle angle

To evaluate the changes in the knee extension angle, preoperative and postoperative knee extension angles were measured in addition to intraoperative knee extension angle. Before TKA, we measured preoperative knee extension angle without anaesthesia. Regarding postoperative knee extension angle, we evaluated the knee extension angle at 6 months after TKA surgery. Preoperative and postoperative knee extension angles were measured using a goniometer, and intraoperative knee extension angles before and after TKA were measured using a navigation system. We evaluated the relationship among extension angles at each time point. We divided all cases into three groups based on the degree of the postoperative knee extension angle at 6 months (full extension group, < 10° postoperative flexion contracture group and ≥ 10° postoperative flexion contracture group) and evaluated the differences in extension angles at each time point among the groups. This study was approved by the Institutional Review Board of our university (Identification Number: 1411020), and written informed consent was obtained from all patients.

### Statistical analysis

At a power of 0.8 and a level of significance of 0.05, this would require at least 34 participants per group according to a power analysis. The number of participants in present study were more than 34 within each group. The Spearman’s rank correlation coefficient (ρ) was used to evaluate the relationship among preoperative, intraoperative, and postoperative knee extension angles. The non-parametric Wilcoxon signed-rank test was used to identify the gender differences. Statistical analyses were performed using JMP, version 14.0 (SAS Institute, Tokyo, Japan).

## Results

**Fig. 2:**
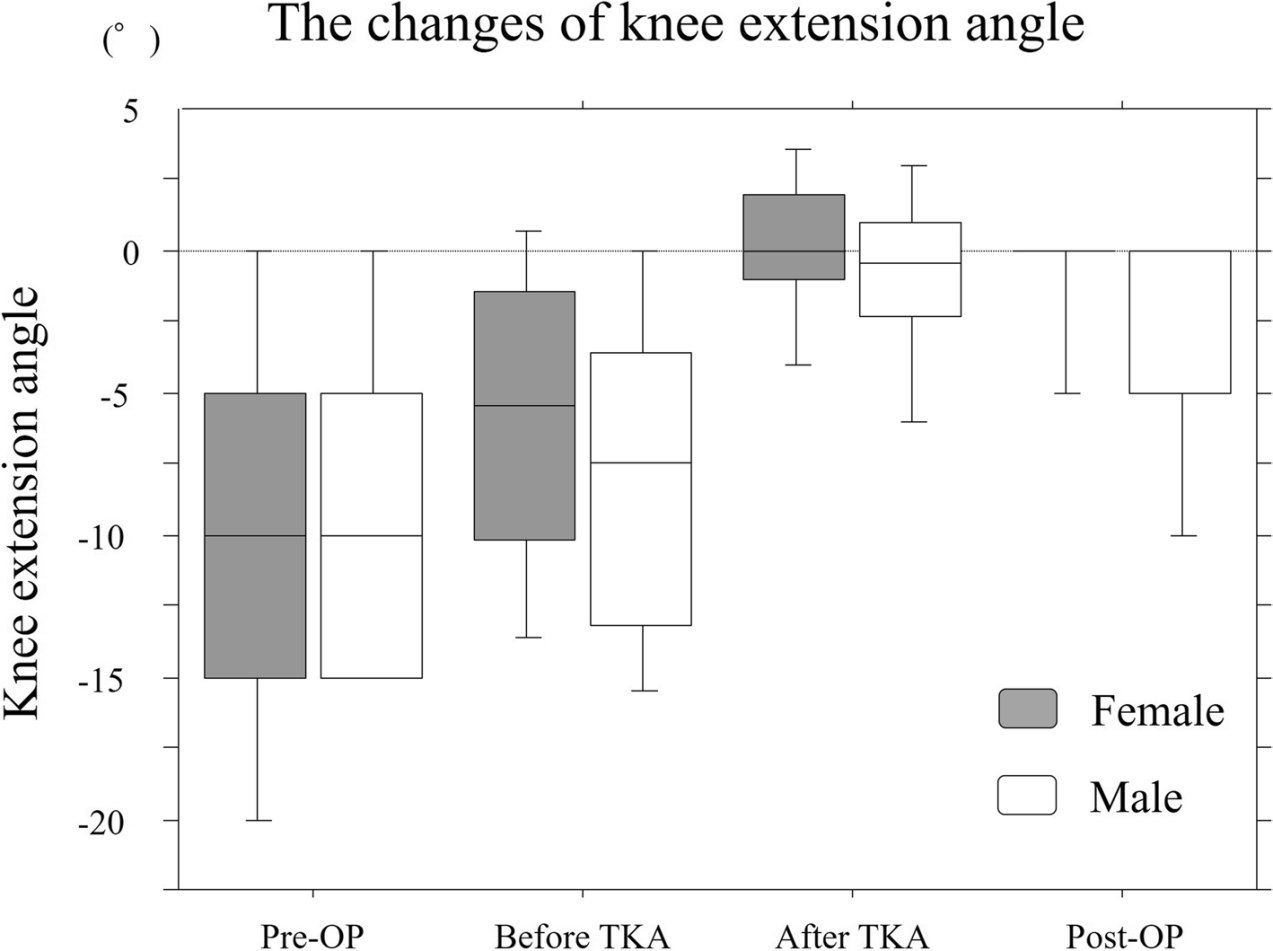
Boxplot and whisker plot of the mean knee extension angle. Pre-OP, preoperative knee extension angle; Before TKA, Intraoperative maximum knee extension angle before TKA; After TKA, Intraoperative maximum knee extension angle after TKA; Post-OP, postoperative knee extension angle at 6 months after TKA

**Fig. 3:**
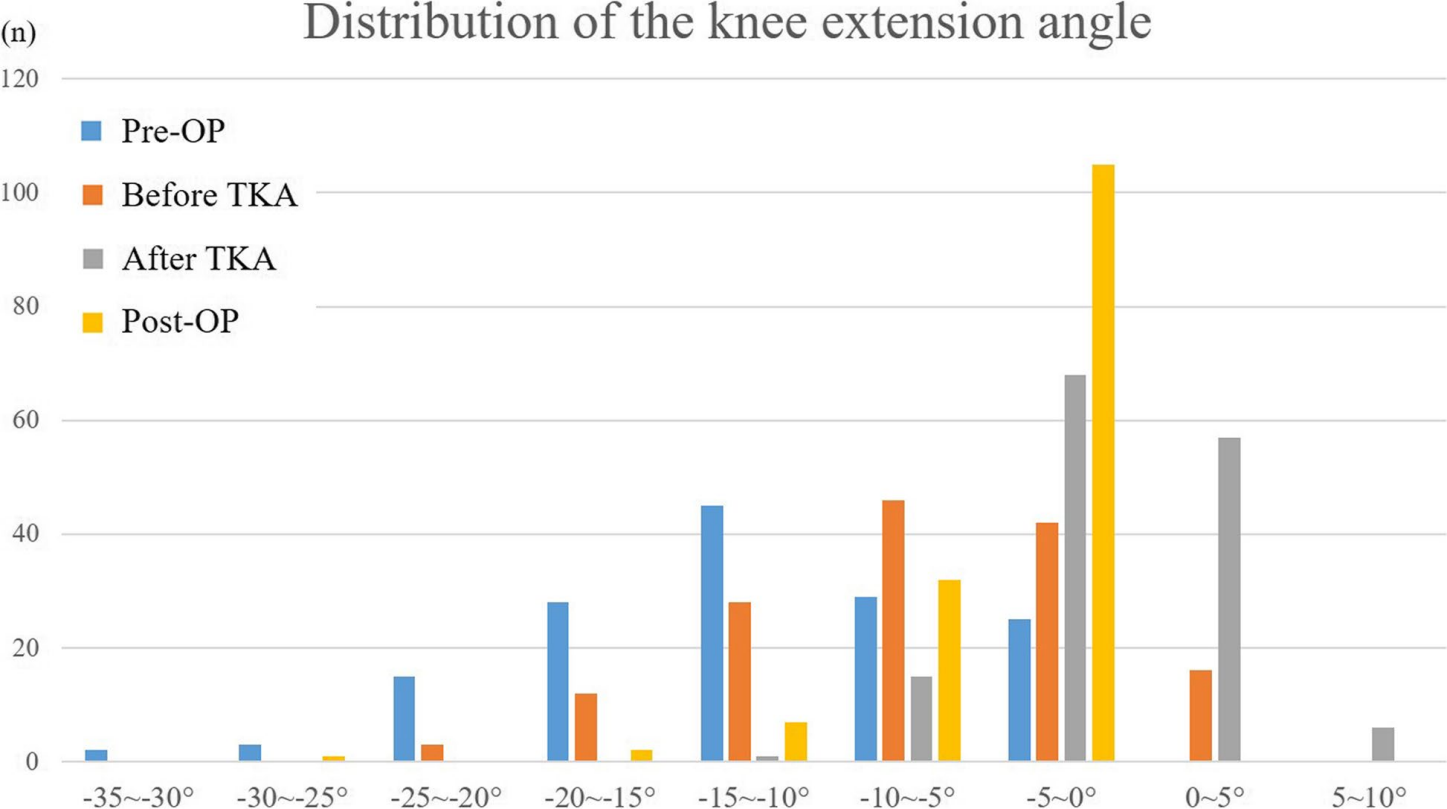
Distribution of preoperative, intraoperative, and postoperative knee extension angles. Pre-OP, preoperative knee extension angle; Before TKA, Intraoperative maximum knee extension angle before TKA; After TKA, Intraoperative maximum knee extension angle after TKA; Post-OP, postoperative knee extension angle at 6 months after TKA

The mean preoperative angle, intraoperative maximum extension angles (before and after TKA), and postoperative extension angle at 6 months after TKA were -9.9°, -6.8°, -0.1° and -2.0°, respectively (Table 3, Fig. 2). Regarding intraoperative maximum extension angle after TKA measured using a navigation system, 58 knees showed ≤ 5° hyperextension and six knees showed > 5° hyperextension. A total of 81% (52 knees in 64 cases) of intraoperative hyperextension cases showed full knee extension at 6 months after TKA. Conversely, at 6 months after TKA, no cases revealed hyperextension and 105 knees accomplished full knee extension. Ten knees showed ≥ 10° flexion contracture, and 32 knees showed < 10° flexion contracture (Fig. 3).

**Table 3:** Mean value of the knee extension angles at each time point

	Total (***n*** = 147)	Male (***n*** = 35)	Female (***n*** = 112)	*p* value
	Mean ± SD	Mean ± SD	Mean ± SD	
Pre-OP	-9.9 ± 6.9°	-9.2 ± 6.8°	-10.1 ± 7.1°	0.48
before TKA	-6.8 ± 5.9°	-8.0 ± 6.2°	-6.3 ± 5.7°	0.17
after TKA	-0.1 ± 3.3°	-0.6 ± 3.4°	0.0 ± 3.3°	0.22
Post-OP	-2.0 ± 3.7°	-3.8 ± 5.7°	-1.4 ± 2.6°	< 0.01**

*Pre-OP* preoperative knee extension angle, *Post-OP* postoperative knee extension angle at 6 months after TKA, *before TKA* Intraoperative maximum knee extension angle before TKA, *after TKA* Intraoperative maximum knee extension angle after TKA, *TKA* total knee arthroplasty

*Significantly different among genders (*p*<0.05)

**Significantly different among genders (*p*<0.01)

Table 4 shows the mean knee extension angle at each time point among the groups classified by postoperative extension angle at 6 months after TKA. The mean intraoperative extension angle after TKA in the postoperative full extension group was 0.4°, showing mild hyperextension. Moreover, the ≥ 10° postoperative flexion contracture group showed significant differences in preoperative extension angle compared to the other groups. Contrarily, regarding intraoperative knee extension angle (before and after TKA), there was no significant difference between the ≥ 10° postoperative flexion contracture group and the < 10° postoperative flexion contracture group.

**Table 4:** Mean knee extension angle changes among three groups classified by a degree of postoperative knee extension angle at 6 months after TKA

	Postoperative full extension group		Postoperative < 10° FC group		Postoperative ≥ 10° FC group	
Sex (n)	**Male**	**Female**	**Male**	**Female**	**Male**	**Female**
	20	85	9	23	6	4
Pre-OP (M/F)	-8.4 ± 6.2°**		-12.0 ± 6.3°*		-19.0 ± 7.7°	
	-7.3 ± 5.7°	-8.6 ± 6.4°	-8.3 ± 6.1°†	-13.4 ± 5.9°	-17.5± 6.1°	-21.3± 9.3°
Before TKA (M/F)	-5.0 ± 5.0°**		-9.7 ± 5.8°		-13.2 ± 4.9°	
	-5.7 ± 5.5°	-4.9 ± 5.0°	-9.1 ± 5.8°	-9.9 ± 5.9°	-13.8 ± 5.0°	-12.4 ± 5.6°
After TKA (M/F)	0.4 ± 3.0°**		-1.6 ± 4.0°		-1.4 ± 2.3°	
	-0.5 ± 3.5°	0.7 ± 2.9°	-0.3 ± 3.9°	-2.0 ± 4.1°	-1.3 ± 2.6°	-1.5 ± 2.3°

*FC*, flexion contracture; *Pre-OP* preoperative knee extension angle, *before TKA* Intraoperative maximum knee extension angle before TKA, *after TKA* Intraoperative maximum knee extension angle after TKA, *TKA* total knee arthroplasty

*Significantly different from the postoperative ≥ 10° FC group (*p*<0.05)

**Significantly different from the postoperative ≥ 10° FC group and < the 10° FC group (*p*<0.05)

† Significantly different from females (*p*<0.05)

**Fig. 4:**
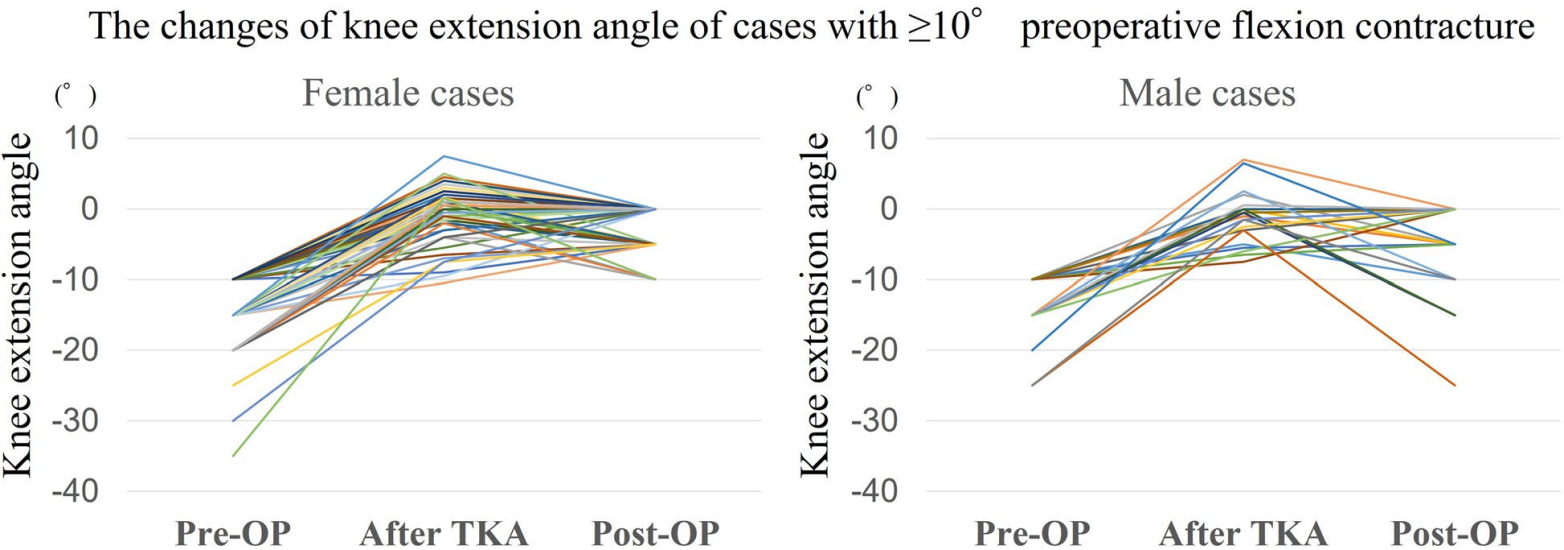
Graph showing changes in the knee extension angle in cases with ≥10° preoperative flexion contracture. Pre-OP, preoperative knee extension angle; After TKA, Intraoperative maximum knee extension angle after TKA; Post-OP, postoperative knee extension angle at 6 months after TKA

Table 5 shows the correlation coefficient among extension angles at each time point. Significantly high correlation coefficients were observed among extension angles at each point. Intraoperative extension angles were correlated with the pre- and postoperative extension angles at 6 months after TKA. Furthermore, we observed gender-specific differences in the knee extension angle changes after TKA. Figure 4 shows the changes in the knee extension angle in cases with ≥ 10° preoperative flexion contracture. Ninety-three cases (21 males and 72 females) showed ≥ 10° preoperative flexion contracture. Among them, six males and four females showed ≥ 10° postoperative flexion contracture, whereas they represented almost full intraoperative extension. Males had a significantly higher flexion contracture rate than females (*p* < 0.01). The intraoperative maximum extension angle after TKA was correlated with the postoperative extension angle at 6 months after TKA only in females. Conversely, the preoperative knee extension angle significantly was correlated with the postoperative extension angle at 6 months after TKA, especially in males (Table 5).

**Table 5:** Correlation coefficient among extension angles

		Total		Males		Females	
		ρ	*p*	ρ	*p*	ρ	*p*
**Pre-OP**	**Before TKA**	0.59	<.0001**	0.59	0.0002**	0.60	<.0001**
**Pre-OP**	**After TKA**	0.27	0.0007**	0.17	0.30	0.31	0.0006**
**Pre-OP**	**Post-OP**	0.35	<.0001**	0.41	0.01*	0.37	<.0001**
**Before TKA**	**After TKA**	0.30	0.0002**	0.29	0.08	0.28	0.003**
**Before TKA**	**Post-OP**	0.41	<.0001**	0.45	0.006**	0.38	<.0001**
**After TKA**	**Post-OP**	0.25	0.0018**	0.05	0.74	0.31	0.0008**

*Pre-OP* preoperative knee extension angle, *Post-OP* postoperative knee extension angle at 6 months after TKA, *Before TKA* Intraoperative maximum knee extension angle before TKA, *After TKA* Intraoperative maximum knee extension angle after TKA

* *p*<0.05, ** *p*<0.01

## Discussion

Our most impressive finding was the gender-specific difference in correlation among knee extension angles at each time point, providing sufficient evidence to argue that the preoperative severe flexion contracture is a risk factor for the postoperative flexion contracture especially in males. Various clinical trials have demonstrated a significant influence of postoperative flexion contracture [12, 23, 29]. Knee extension angle can influence knee extensor strength, and intervention for knee extension range improvement might help improve knee extensor performance [27]. Moreover, the quadriceps burden for stabilising knees increases as flexion contracture worsens [25]. Although surgical methods to avoid flexion contracture have been reported [17, 22, 24], previous reports suggested that preoperative severe flexion contracture increased the risk of developing stiffness afterward even if deformity is corrected during TKA surgery [19]. Our findings are congruent with those of this paper in that the preoperative knee extension angle was found to influence postoperative knee extension angle significantly. Another study assessed the importance of rehabilitation, stating that the maintenance of full extension should be focused on during the early rehabilitation phase after surgery [31]. Some of our patients showed postoperative flexion contracture despite intraoperative full knee extension. These results might be derived from the postoperative rehabilitation in addition to the preoperative flexion contracture influence. Moreover, other researchers reported improved postoperative flexion contracture for up to 2 years, demonstrating the importance of postoperative rehabilitation duration [28]. Combining the observations from these reports and our results, we argue that the intraoperative optimal knee extension angle and postoperative rehabilitation are required to maintain full extension after TKA.

Regarding the measurement of knee angle, previous studies used X-rays and a goniometer after TKA surgery [18, 28]. In contrast, we evaluated intraoperative knee extension angle using a navigation system. We could measure every 0.5° of knee extension angle intraoperatively due to the precision of the navigation system. Using this method, not a few cases showed mild hyperextension in this study. Many researchers have discussed the negative effect of hyperextension on clinical results. Hyperextension of a knee joint caused postero-lateral corner damage [4], and TKA with postoperative hyperextension showed worse functional outcomes at mid-term follow-up [4, 20]. Although 44% of our cases (64 knees) showed intraoperative mild hyperextension under passive extensional stress after TKA measured using a navigation system, no cases showed hyperextension at 6 months after TKA. Nearly all cases among those (58 knees) were < 5° intraoperative hyperextension, which is undetectable without a navigation system. With the high acquisition rate of postoperative full extension at 6 months in the intraoperative hyperextension cases, the results suggested that intraoperative < 5° hyperextension measured using a navigation system is acceptable to accomplish the optimal postoperative knee extension angle. Several of our cases represented intraoperative > 5° hyperextension. However, these cases were few in number to be able to assess the clinical effect. Therefore, further research is necessary to determine the permissible hyperextension.

We observed gender-specific differences in knee extension angle changes. Previous studies reported many anatomic differences between the knees of males and females [15]. Among them, our results may be derived from gender-specific differences in soft tissue properties. Knee ligaments have more laxity under passive stress in females than in males [1, 26]. Hsu et al. investigated the cadaver knee rotational laxity, reporting a lower torsional joint stiffness and higher rotatory joint in female knees than in male knees responding to combined rotatory loads [11]. Shultz et al. mentioned that sex hormonal changes mediate knee laxity changes across the menstrual cycle [32]. Similarly, hormones including oestrogen were proven to affect gender-related differences in the ligament damage frequency in anterior cruciate ligament injuries [6]. In addition, Hansen et al. suggested that the oestradiol level elevation might affect tendon morphology and biomechanical properties [8]. In line with previous studies, hormones may have led to gender-specific differences in knee extension angle changes. We performed TKA using the same surgical procedure and no differences were observed in the pre- and intraoperative knee extension angles between sexes. However, males showed a significantly higher flexion contracture recurrence rate, which could be due to the muscle strength elasticity and soft-tissue characteristics. Moreover, such gender-specific differences in the soft-tissue properties might influence the effect of anaesthesia wearing off on knee extension angle changes. Hence, male patients with preoperative flexion contracture need more attention and the appropriate postoperative rehabilitation protocol.

The strengths of this study include the use of a navigation system, which enabled a precise evaluation of the extension angle. Another advantage is the intraoperative evaluation of patients undergoing TKA. No previous studies have shown the influence of the intraoperative knee extension angle under anaesthesia measured using a navigation system on the postoperative knee extension angle. Moreover, the same coordinate axis was used for knee extension angle measurement using a navigation system before and after TKA. This procedure enabled us to compare between the pre- and postoperative statuses of the knee more accurately. We measured the intraoperative knee extension angle before TKA with joint capsular closure and the intraoperative knee extension angle after TKA under anaesthesia after skin closure. The temporal change of the intraoperative knee extension angle measured using a navigation system has not been previously assessed. Hence, this study is novel in considering the optimal intraoperative knee extension angle in TKA.

The fundamental limitation of our study includes the measurement method difference, with the intraoperative measurement of knee extension angle using a navigation system and the pre- and postoperative measurements using a goniometer. Moreover, our study exclusively included patients with varus-type arthritis. The different rehabilitation terms and methods possibly affected flexion contracture recurrence. Finally, the procedure involved passive knee extensional stress by a single surgeon. Although the ICCs were sufficiently high, the stress force was not standardised. Further research is needed to determine the influence of intraoperative maximum knee extension angle on postoperative clinical results.

## Conclusion

Our results revealed that intraoperative knee extension angle under anaesthesia significantly correlated with pre- and postoperative knee extension angles in TKA, and showed that intraoperative ≤ 5° hyperextension measured using a navigation system is acceptable for postoperative proper knee extension angle in TKA. Moreover, preoperative severe flexion contracture is a risk factor for postoperative flexion contracture despite accomplishing intraoperative full knee extension, especially in males.

## Data Availability

Available.

## Abbreviations

TKA: Total knee arthroplasty
FC: Flexion contracture
ROM: Range of motion
PS: Posterior-stabilised
BCS: Bi-cruciate-stabilised
ICCs: Intraclass correlation coefficients
